# Sleeve gastrectomy attenuates high fat diet-induced non-alcoholic fatty liver disease

**DOI:** 10.1186/s12944-018-0875-5

**Published:** 2018-10-24

**Authors:** Erli Pei, Yang Liu, Weiqing Jiang, Songruo Lin, Lei Huang, Moubin Lin, Li Cai

**Affiliations:** 1 Department of General Surgery, Yangpu Hospital, Tongji University School of Medicine, Shanghai, 200433 China; 2 Department of Gerontology, Tongji Hospital, Tongji University School of Medicine, Shanghai, 200065 China; 3 Department of Science and Research, Tongji Hospital, Tongji University School of Medicine, Shanghai, 200065 China

**Keywords:** Non-alcoholic fatty liver disease, Sleeve gastrectomy, High-fat diet, Fibroblast growth factor 21

## Abstract

**Background:**

A high-fat diet (HFD) is known to lead to obesity, and contributes to the progression of non-alcoholic fatty liver disease. The objective of this study was to evaluate the effects of sleeve gastrectomy (SG) on the progression of HFD-induced hepatic steatosis.

**Methods:**

Fifteen 4-week-old, male Wistar rats were randomly assigned into three groups: NC, HFD + SHAM and HFD + SG. Their body weight, glucose-lipid metabolism, inflammation indices, hepatic steatosis and fibroblast growth factor 21 (FGF21) levels were measured.

**Results:**

Postoperatively, body weights in the HFD + SHAM and HFD + SG group rats decreased during the first week. Thereafter, HFD + SG rats regained their body weight. Differences in insulin, homeostasis model assessment of insulin resistance, triglyceride, free fatty acid, tumor necrosis factor-α and monocyte chemotactic protein-1 levels were statistically significant across the three groups (all *P* < 0.05). Interestingly, FGF21 levels in the HFD + SG group were markedly lower than in the HFD + SHAM group (*P* = 0.015), however, there were no differences in the NC group. Hematoxylin and eosin staining demonstrated that more vacuoles were present in the HFD + SHAM liver when compared to the HFD + SG liver. Oil-red O staining showed less red dots in the HFD + SG liver.

**Conclusions:**

Despite eating, surgical re-routing of the gut may prevent weight accumulation, regulate glucose-lipid metabolism and insulin sensitivity, control a chronic inflammatory state, change the secretion pattern of FGF21 and alleviate the severity of fatty liver.

## Background

The rate of morbid obesity, defined by a body mass index ≥40 kg/m^2^, is expanding worldwide. In the last three decades, rates of being overweight or obese have increased 27.5% for adults, and 47.1% for children [1]. Genetics, social economy, cultural influences, and other predisposing factors are known to impact the development and progression of obesity [2]. One significant characteristic of obesity is the high occurrence of comorbidities, including coronary heart disease, type 2 diabetes mellitus and non-alcoholic fatty liver disease (NAFLD) [3].

Bariatric surgery is an established treatment, which is part of complex and interdisciplinary therapeutic approach, for patients with severe obesity and metabolic syndrome [4], allowing for long term weight loss [5, 6]. Bariatric surgery not only reduces long-term mortality, but also improves the histopathological aspects of NAFLD [7, 8]. However, the exact mechanisms underlying this have not yet been elucidated.

The main histological characteristic of NAFLD is hepatic lipid accumulation/steatosis [9]. The exposure and overload of fatty acids harm hepatocytes via the intracellular accumulation of lipid intermediates, which is defined as lipotoxicity [10]. This hepatic lipid accumulation and intracellular stressors activate the transcription and release of pro-inflammatory factors, including interleukin (IL)-6 and tumor necrosis factor (TNF)-α [11]. The elevation in circulating levels of pro-inflammatory cytokines, and reduced anti-inflammatory factors, result in a chronic, low-grade inflammatory state that is recognized as an important pathogenic mechanism of NAFLD [11, 12]. Furthermore, this altered lipid metabolism is believed to be a central mechanism in the development of insulin resistance, by activating different kinases [13].

Fibroblast growth factor 21 (FGF21), a member of the fibroblast growth factor family, has emerged as a leading candidate in the regulation of energy homeostasis, glucose-lipid metabolism and insulin sensitivity [14, 15]. FGF21 binds to β-klotho and fibroblast growth factor receptors (FGFRs) and induces the dimerization and autophosphorylation of the FGFR [16]. Activated FGFRs then initiate their biological functions. Mouse tissue is reported to express FGF21 in regions including the liver, adipose tissue [17, 18] and the acinar pancreas [19]. Generally, circulating FGF21 is largely derived from the liver, and circulating levels correlate well with hepatic expression [20]. The administration of FGF21 reverses hepatic steatosis, prevents diet-induced obesity, and alleviates insulin resistance and dyslipidemia [15, 21].

Whilst bariatric surgery is effective in over 75% of patients, revisional surgeries are required in up to 20% of patients, and the reasons underlying suboptimal outcomes is currently unclear [22]. One possibility is that patients who require revisional surgery are not following the recommendations regarding caloric and macronutrient intake. Recent studies suggest that there is little, if any, reduction in dietary fat intake in the months and years following surgery [23, 24]. With these data in mind, we hypothesized that bariatric surgery would alter gut physiology, and prevent the expansion of HFD-induced hepatic steatosis in obese rats. To test this hypothesis, we performed bariatric surgery on obese rats, maintained the animals on a HFD, and assessed the clinical diagnostic criteria of NAFLD as well as cellular changes in FGF21.

## Methods

### Animals

Fifteen 4-week-old, male, Wistar rats weighing 52–65 g were purchased from the Laboratory Animal Center of Shandong University (Shandong, China). The rats were individually housed in ventilated cages with a natural light/dark cycle, at a constant ambient temperature (24 °C–26 °C) and humidity (50 ± 2%). After a five-day period of adaptation to laboratory conditions, rats were divided into three groups: normal chow (NC), high fat diet without sleeve gastrectomy (HFD + SHAM) and HFD with sleeve gastrectomy (HFD + SG). The normal diet consisted of 10% kcal from fat (D12450B diet, Research Diets Inc., New Brunswick, NJ, USA), whereas the HFD consisted of 60% kcal from fat (D12492 diet, Research Diets Inc.).

### Surgical procedures

Halfway through the experiment (3 months), animals were fasted for 12 h, weighed, anesthetized (4% sevoflurane; RWD Co., Shanghai, China), and placed in the supine position on a surgical board with their extremities immobilized. For rats in the HFD + SG group, the greater curvature from the antrum to the fundus across the forestomach and glandular stomach was incised and ~ 90% of the forestomach and 70% of the glandular stomach were removed. The incision line in the stomach was then closed using three layers of polydioxanone sutures. For rats in the HFD + SHAM group, laparotomy was performed to expose the stomach, esophagus, and small intestine. No other procedure was carried out. Furthermore, operative time was prolonged to induce a comparable degree of anesthetic stress experienced by the HFD + SG rats. Following surgery, rats in the HFD + SHAM and HFD + SG groups received a HFD for 42 d, whereas rats in the control group received normal chow.

### Collection of blood and liver samples

Forty-two d following the operations, the rats fasted overnight. Blood samples were collected from the hearts of rats into chilled EDTA tubes containing a dipeptidyl peptidase IV inhibitor. After centrifugation (1000×g) at 4 °C for 15 min, the supernatant was immediately collected. The samples were stored at − 80 °C for future analysis. Liver tissue samples were taken at the time of surgery. For histochemical examination using hematoxylin and eosin (H&E) staining, liver samples were embedded into paraffin and were cut into 6 μm-thick slices. Staining was performed using a commercial kit (#3500, BBC Biochemical, Atlanta, GA, USA). The size of adipocytes was determined with an optical microscope (ECLIPSE Ti, Nikon, Japan) using an NIS-Elements imaging platform purchased from Nikon Instruments Inc. (Melville, NY, USA). For Oil-red O staining, liver samples were frozen in liquid nitrogen and sectioned at 8 μm in thickness using a Cryostat. These sections were stained by using Oil-red O (Electron Microscopy Sciences, Hatfield, PA) for 30 min.

### Laboratory measurements

Postoperatively, the body weights were monitored weekly. Serum lipid profiles, including total cholesterol, triglyceride and free fatty acid (FFA) were measured using enzyme-linked immunosorbent assay (ELISA, R&D Systems, Minneapolis, Minnesota, USA).

Plasma insulin was quantified using ELISA, in accordance with the manufacturer’s instructions (R&D Systems). Prior to operating, and at 2 and 8 weeks postoperatively, homeostasis model assessment of insulin resistance (HOMA-IR) was calculated to evaluate insulin resistance according to the following formula: HOMA-IR = fasting insulin (mIU/L) × fasting glucose (mmol/L)/22.5.

Plasma levels of IL-6, IL-1β, monocyte chemotactic protein-1, TNF-α and FGF21 were assayed with ELISA, in accordance with the manufacturer’s instructions (R&D Systems).

### Statistical analysis

Results are presented as mean ± the standard error. The distribution of continuous variables was assessed for normality using Levene’s test. Differences between means were analyzed using one-way Analysis of Variance combined with Bonferroni correction test (*P* = 0.05/3). All statistical analyses were two sided, and *P* < 0.05 was considered statistically significant. All statistical analyses were performed using Statistical Analysis System software (version 9.1.3, SAS Institute, Cary, NC, USA).

## Results

### Body weights

**Fig. 1:**
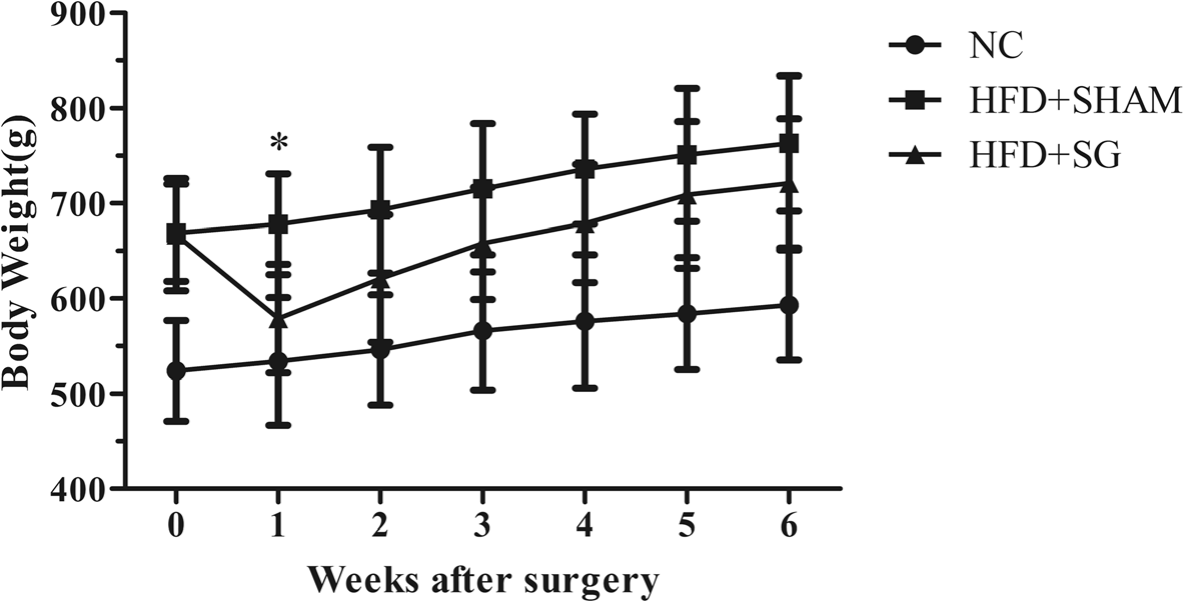
Body weight of rats 6 weeks after surgery. Data are mean ± standard deviation. *P < 0.05

Postoperatively, body weights in the HFD + SHAM and HFD + SG group rats decreased over the course of the first week (*P* = 0.021, Fig. 1). Thereafter, HFD + SG rats regained their body weight, but at a higher rate compared to the HFD + SHAM rats.

### Glucose-lipid metabolism

Briefly, differences in insulin levels across the three groups was statistically significant (*P* = 0.033, Table 1). Based on HOMA-IR calculations, the HFD + SG group rats became significantly more insulin sensitive compared to the HFD + SHAM group (*P* = 0.021, Table 1).

**Table 1:** Effect of high fat diet on glucose-lipid metabolism and inflammation across the three groups

Variables	NC	HFD + SHAM	HFD + SG	*P*
total cholesterol (mmol/L)	6.57 ± 0.14	7.07 ± 0.30	6.85 ± 0.65	0.177
triglyceride (mmol/L)	1.66 ± 0.42	2.38 ± 0.26	2.10 ± 0.38	*0.027*
free fatty acid (μmol/L)	472.96 ± 61.27	737.76 ± 179.86	716.50 ± 197.11	*0.039*
Insulin (mIU/L)	44.9 ± 6.1	47.7 ± 6.5	36.86 ± 4.9	*0.033*
homeostasis model assessment of insulin resistance	10.62 ± 1.63	11.75 ± 2.14	8.71 ± 1.01	*0.039*
tumor necrosis factor-α (pg/mL)	270.03 ± 66.37	378.53 ± 25.34	317.31 ± 72.94	*0.037*
monocyte chemotactic protein-1 (pg/mL)	496.97 ± 38.16	634.29 ± 49.93	522.85 ± 65.12	*0.003*
interleukin-6 (pg/mL)	93.15 ± 12.70	96.37 ± 15.60	79.09 ± 29.58	0.419
interleukin-1β (pg/mL)	18.15 ± 5.32	13.11 ± 3.66	19.39 ± 6.87	0.203
fibroblast growth factor 21 (pg/mL)	103.29 ± 9.88	165.35 ± 29.24	112.43 ± 16.64^*^	*0.001*

Data are mean ± standard deviation. ^*^P < 0.016, vs. HFD + SHAM

Triglyceride levels in the NC, HFD + SHAM and HFD + SG groups were 1.66 ± 0.42 mmol/L, 2.38 ± 0.26 mmol/L and 2.10 ± 0.38 mmol/L, respectively (*P* = 0.027, Table 1). FFA levels in the NC, HFD + SHAM and HFD + SG groups were 472.96 ± 61.27 mmol/L, 737.76 ± 179.86 mmol/L and 716.50 ± 197.11 mmol/L, respectively (*P* = 0.039, Table 1). However, there were no statistically significant differences between the HFD + SHAM and HFD + SG groups (both *P* > 0.05).

### Inflammation

Differences in TNF-α and monocyte chemotactic protein-1 levels across the three groups were statistically significant (both *P* < 0.05, Table 1).

### Hepatic steatosis

**Fig. 2:**
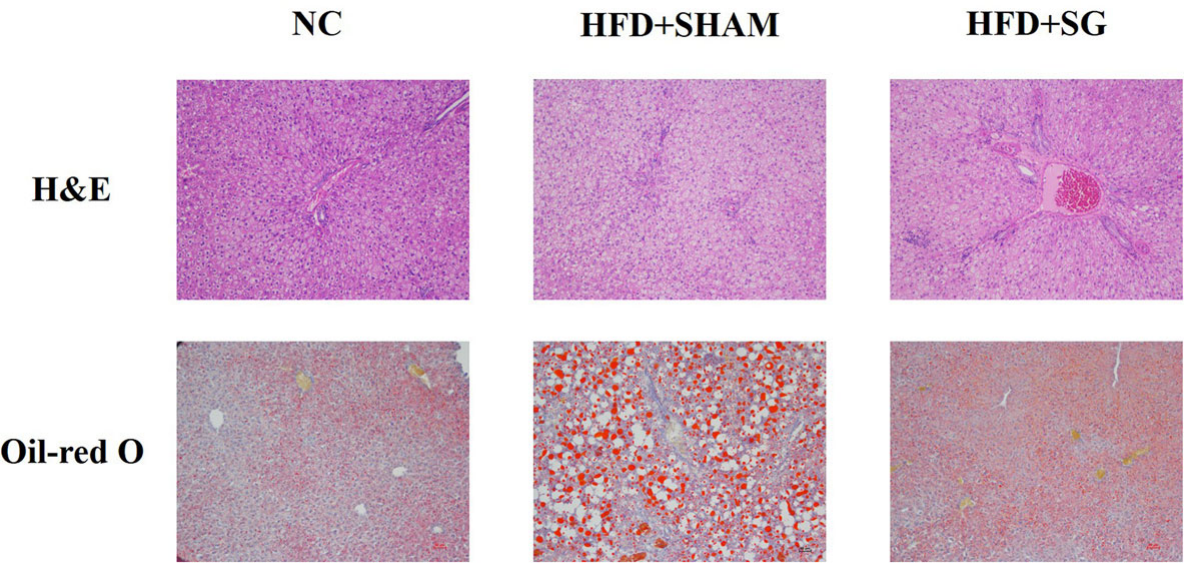
Representative images of hematoxylin and eosin staining and Oil-red O staining of liver slice in three groups

H&E staining showed that more vacuoles were present in the HFD + SHAM liver compared to the HFD + SG liver. Consistently, Oil-red O staining showed less red dots in the HFD + SG liver (Fig. 2).

### FGF21

**Fig. 3:**
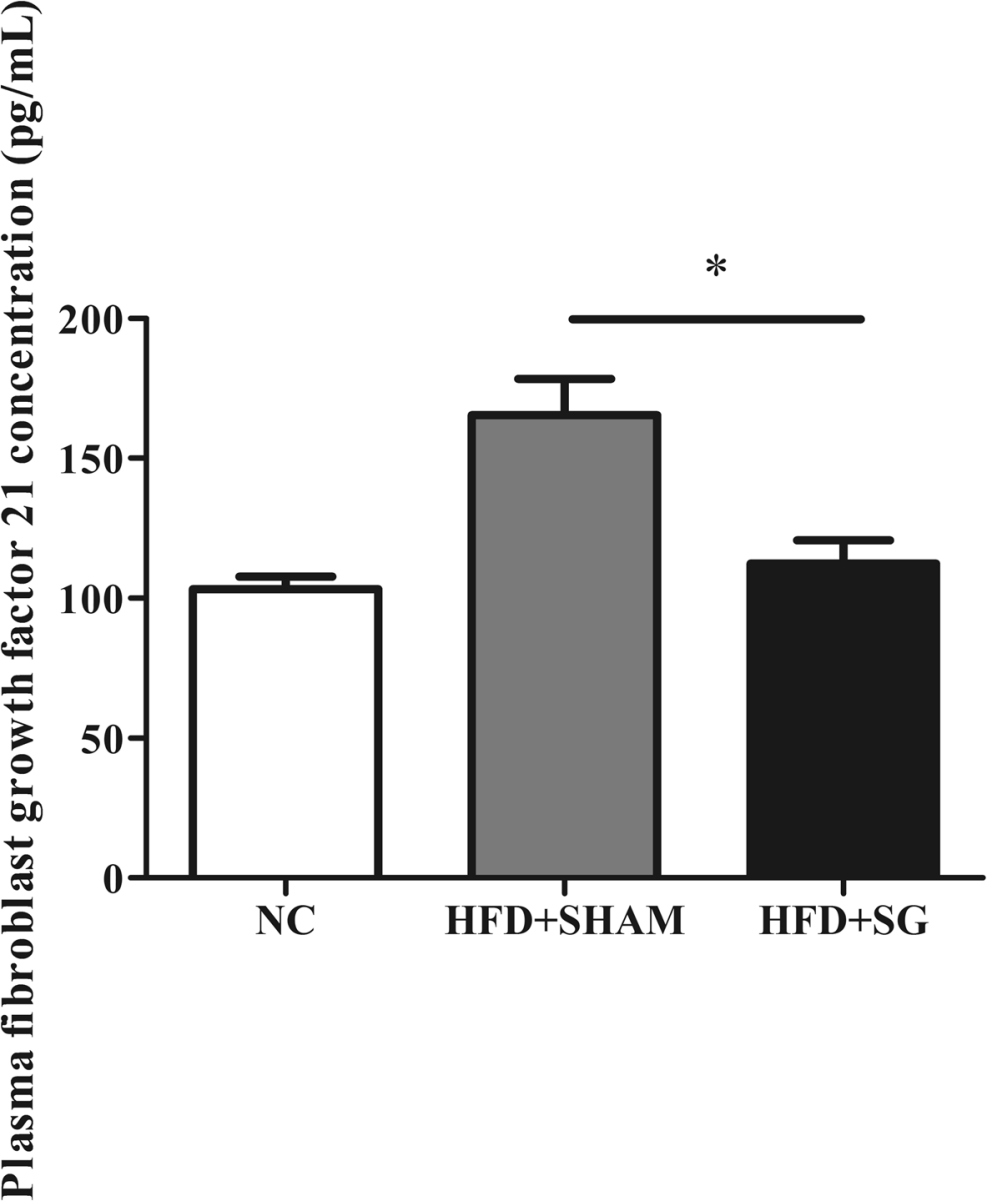
Plasma fibroblast growth factor 21 cconcentration in three groups. Data are mean ± standard deviation. *P < 0.05

Interestingly, FGF21 levels were markedly lower in the HFD + SG group, compared to the HFD + SHAM group (*P* = 0.015, Table 1). However, there was no difference observed in the NC group (Table 1, Fig. 3).

## Discussion

In this study, we investigated the effect of bariatric surgery on NAFLD progression in obese rats maintained on a HFD, which is known to produce metabolic dysfunction in the liver. We found that despite eating, bariatric surgery could prevent weight accumulation, regulate glucose-lipid metabolism and insulin sensitivity, and control a chronic inflammatory state in this rat model of bariatric surgery. Additionally, FGF21 levels in the HFD + SG group were markedly lower than in the HFD + SHAM group. H&E staining and Oil-red O staining suggested that the severity of fatty liver had been alleviated after surgery. These data suggest that bariatric surgery can relieve risk factors known to influence the development of NAFLD in HFD rats.

FGF21 phosphorylates Akt and ameliorates insulin resistance in peripheral tissues [25]. Reduced insulin resistance decreases ChREBP-mediated de novo lipogenesis by promoting glucose utilization in the liver, and by inhibiting hepatic gluconeogenesis, decreasing the accumulation of lipids [26, 27]. Interestingly, FGF21 gene transfer has been shown to significantly reduce hyperinsulinemia and attenuate insulin resistance in mice fed a HFD, leading to significant improvements in glucose tolerance [28]. Additionally, FGF21 infusion has been demonstrated to improve insulin responsivity in wild-type, and FGF21 gene transfer mice [21, 28]. This effect may be explained by the insulin-independent activity of FGF21 in enhancing glucose uptake in muscle and adipose tissue [29], through the activation of ERK1/ERK2, and through the induction of glucose transporter-1 expression [30, 31]. Furthermore, systemic administration of FGF21 results in the amelioration of glucose and lipid parameters in diabetic rodents, as well as nonhuman primates [15, 16, 32, 33].

FGF21 possesses potent activity in enhancing lipid oxidation as well as suppressing triglyceride synthesis [30, 34]. A recent study showed that FGF21 limits FFA accumulation by potentiating the activation of long chain fatty acids to acyl-CoA, and shunting them towards mitochondrial β-oxidation. This leads to attenuated hepatic steatosis and diminishes the lipotoxic effects [35]. Moreover, FGF21 has been identified as a key modulator for adiponectin secretion in white adipose tissue, which stimulates the deacetylation of ceramides and decreases lipotoxicity [36, 37]. Daily injections of FGF21 inhibited the expression of a battery of genes involved in fatty acid and triglyceride synthesis, resulting in a dramatic reduction in hepatic triglyceride levels [15]. Similarly, Gao et al. observed an acute effect of FGF21 gene transfer in the livers of lean mice, and in mice fed a high-fat diet, showing a marked attenuation in the expression of multiple key genes involved in lipogenesis, resulting in a corresponding alleviation in fatty liver [28]. FGF21 overexpression reverses the upregulated expression of SREBP-1c and FAS, two key enzymes required for lipid synthesis, in fatty acid-treated human liver-derived HepG2 cells [34].

A recent study revealed a novel functional interaction between the FGF21 system and proinflammatory signaling involved in the repressive effects of TNF-α on the expression of β-Klotho, a pivotal molecule in the cellular machinery that mediates the response to FGF21 [38]. It is known that FGF21 also counteracts the negative effects of TNF-α on adiponectin secretion [37]. The complex cross-talk between the FGF21 system and inflammatory pathways is also highlighted by the fact that FGF21 is capable of inhibiting NF-κB activity [39], is induced by inflammatory stimuli, and protects animals against the toxic effects of lipopolysaccharide [40]. In line with these above studies, our data showed that after surgery, rats in the HFD + SG group had lower FGF21 levels (Table 1, Fig. 3). Interestingly, we also observed an improvement of body weight, glucose-lipid metabolism and insulin sensitivity, and inflammatory state (Table 1). Additionally, H&E staining and Oil-red O staining suggested that the severity of fatty liver had been alleviated after surgery (Fig. 2).

As noted previously, the treatment of obese mice with FGF21 normalizes liver function, and reduces fibrosis and inflammation. Similarly, it was recently reported at the 2017 Meeting of the European Association for the Study of Liver Disease that pharmacological treatment with FGF21 reduces fatty liver. In humans, FGF21 serum levels correlate with obesity and importantly appear to reflect the degree of fatty infiltrations in the liver, suggesting that levels could serve as a marker for NAFLD [41]. Despite these beneficial effects, the application of FGF21 in clinical practice is limited by its short half-life, which is less than 2 h in mice [42], and potential toxicity in skeletal homeostasis [43, 44].

There are some limitations in this study. Primarily, the diets were freely available to all the mice in each group, so the intake of each individual mouse may have been unequal. Additionally, data from different mouse strains are highly variable due to different genetic backgrounds and lifestyles. Therefore, these results must be confirmed in other animal models.

## Conclusion

In summary, our data are consistent with our original hypothesis that despite eating, surgical re-routing of the gut can prevent weight accumulation, regulate glucose-lipid metabolism and insulin sensitivity, control a chronic inflammatory state, change the secretion of FGF21 and alleviate the severity of fatty liver. Further studies on this therapy are required to elucidate the molecular and cellular basis underlying NAFLD progression in obesity. Larger, well-designed studies, including a diverse group of animals, should be performed to validate these results and expand the understanding of the biological mechanisms involved in the association between FGF21 and NAFLD progression.

## Abbreviations

ELISA: Enzyme-linked immunosorbent assay
FFA: Free fatty acid
FGF21: Fibroblast growth factor 21
FGFR: Fibroblast growth factor receptors
H&E: Hematoxylin and eosin
HFD: High-fat diet
HOMA-IR: Homeostasis model assessment of insulin resistance
IL: Interleukin
NAFLD: Non-alcoholic fatty liver disease
SG: Sleeve gastrectomy
TNF: Tumor necrosis factor
